# Exosomes in Breast Milk: Their Impact on the Intestinal Microbiota of the Newborn and Therapeutic Perspectives for High-Risk Neonates

**DOI:** 10.3390/ijms26073421

**Published:** 2025-04-05

**Authors:** Delia Cristóbal-Cañadas, Rocio Parrón-Carrillo, Tesifón Parrón-Carreño

**Affiliations:** 1 Neonatal Intensive Care Unit, Torrecárdenas University Hospital, 04009 Almería, Spain; dcc380@ual.es; 2 Department of Psychology, Faculty of Health Sciences, University of Almeria, 04120 Almería, Spain; rocio.parron@hotmail.com; 3 Department of Nursing, Physiotherapy and Medicine, University of Almería, 04120 Almería, Spain

**Keywords:** exosomes, breast milk, intestinal microbiota, neonates, necrotising enterocolitis

## Abstract

Breast milk exosomes are essential for the nutrition and immune development of the newborn. These 30–150 nm extracellular vesicles contain microRNAs (miRNAs), mesessenger RNAS (mRNA)s, proteins and lipids that facilitate cellular communication and modulate the neonatal immune system. In this article, we analyse the impact of breast milk exosomes on the intestinal microbiota of the newborn, especially in high-risk neonates such as preterm infants or neonates at risk of necrotising enterocolitis (NEC). Exosomes promote the colonisation of beneficial bacteria such as *Bifidobacterium* and *Lactobacillus* and strengthen the intestinal barrier. They also regulate the immune response, balancing defence against pathogens and tolerance to non-pathogenic antigens. This effect is key for high-risk infants, who benefit from their anti-inflammatory and preventive properties against complications such as NEC. Research points to their potential therapeutic uses in neonatal care, opening up new opportunities to improve the health of vulnerable newborns through the protective effects of breast milk exosomes.

## 1. Introduction

Breast milk (BM), with its unique combination of essential nutrients and bioactive factors, is the gold standard for feeding preterm and preterm infants [1]. Breastfed infants have significant health benefits compared to formula-fed infants [2]. Unlike formula milk, whose composition is standardised within a very narrow range, the molecular composition of breast milk is dynamic and changes during feeding, sleep–wake cycles and lactation. The composition of this milk provides information about social and environmental factors beyond mere nutrition, and forms a molecular link between mother, infant and the environment, so that the molecular composition is unique to each mother [1]. In addition to being a source of nutrients, breast milk contains a number of biologically active components such as immunoglobulins, cytokines, chemokines, growth factors, hormones, lactoferrin and even the maternal microbiota, which contribute to the maturation of the different biological systems of the infant, whose bioactive compounds vary in composition during different breastfeeding episodes [3].

The composition of breastmilk is neither static nor immutable. It varies with feeding, time of day and phase of lactation. Maternal and environmental factors, geographical variations and gestational age also affect the composition of breast milk [4]. Finally, the storage, handling and method of administration of infant milk can directly influence its stability and bioactivity [5]. Among the variety of bioactive components, microRNAs are included [6]. Elevated levels of miRNAs have been detected in body fluids such as the plasma, urine, saliva, seminal fluid, tears, cerebrospinal fluid (CSF) and, more recently, in milk [7]. miRNAs are highly conserved small non-coding RNAs of 19–24 nucleotides that play critical and essential roles in the modulation of gene expression through their interaction with cellular mRNAs [8]. miRNAs are widely present in many human body fluids [7]. Breast milk appears to be one of the richest sources of microRNAs, which is also highly conserved in its different fractions [6]. Human breast tissue has a specific miRNA expression profile, and miRNAs in breast milk mainly come from the mammary gland epithelium and not from the maternal circulation [9]. They are then thought to enter the systemic circulation of the fed infant and exert the tissue-specific epigenetic regulation of several functions, including immunoprotection and development [10].

Breast milk is much more than a source of nutrients for the newborn; it is a complex biological fluid containing many bioactive components that contribute significantly to the immune and microbiological development of the infant. Among these components are exosomes, extracellular vesicles between 30 and 150 nm in diameter that play a crucial role in cell-to-cell communication by transporting miRNA, mRNA, proteins and lipids. These exosomes are considered to be an essential bridge between the mother and infant, facilitating immune modulation and contributing to the development of the neonatal gut microbiome [3].

The present review focuses on exploring the recent literature on exosomes in breast milk, their impact on the neonatal gut microbiota and their potential therapeutic use in high-risk neonates, such as preterm and necrotising enterocolitis (NEC)-prone neonates.

## 2. Compositions and Functions of Exosomes in Breast Milk

Breast milk exosomes contain a number of bioactive biomolecules that play a key role in cell-to-cell communication and the regulation of immune functions in the newborn. The discovery 10 years ago that human breast milk contains abundant extracellular vesicles has attracted considerable attention in this field [11]. These vesicles consist of a lipid bilayer that includes a number of components, such as miRNA, mRNA, proteins and lipids. These molecules are transported from the mammary gland cells to the neonatal gut, where they exert their effects [12,13].

Several studies investigating miRNAs in breast milk exosomes have shown that they are enriched in several biological functions, such as the actin cytoskeleton, glycolysis and gluconeogenesis, aminoacyl-tRNA biosynthesis, the phosphate–pentose pathway, the regulation of galactose metabolism and fatty acid synthesis as well as in several immunological pathways [14,15]. The miRNAs present in breast milk exosomes are able to regulate the expression of key genes in neonatal intestinal cells, directly affecting intestinal barrier maturation and immune responses. Recent studies have identified more than 600 miRNAs in breast milk exosomes, many of which are associated with the regulation of immune and inflammatory processes [13]. In addition, exosomes also contain proteins and lipids that play important roles in antimicrobial function and the modulation of the immune response, promoting interaction with intestinal cells and favouring a healthy environment for microbiota development [16]. Moreover, proteomic analysis of extracellular vesicles (Evs) in human milk has shown that many of the proteins identified are derived from immune cells [17]. Surprisingly, a significant proportion of these proteins had not previously been detected in breast milk, suggesting that the study of EV loads may contribute to the discovery of new biomarkers and functions for future studies.

## 3. Impact on Newborn Intestinal Microbiota

The human microbiome is a vast system of microbial communities that live in different parts of the human body and play a fundamental role in human physiology and health. Components of the gastrointestinal microbiota help to establish mucosal barrier function and nutrient absorption, produce metabolites, contribute to the metabolism of xenobiotics, enhance the immune system and prevent colonisation by pathogens [18]. The intestinal colonisation of infants is thought to begin at birth, when they come into contact with the microbiota of the mother’s vagina and faeces. This intestinal colonisation is further modulated by exposure to colostrum and breast milk, formula and other foods, as well as skin and environmental microbial exposure [19]. This early development plays an important role in the development of the intestinal barrier and the maturation of the immune system [20].

**Figure 1:**
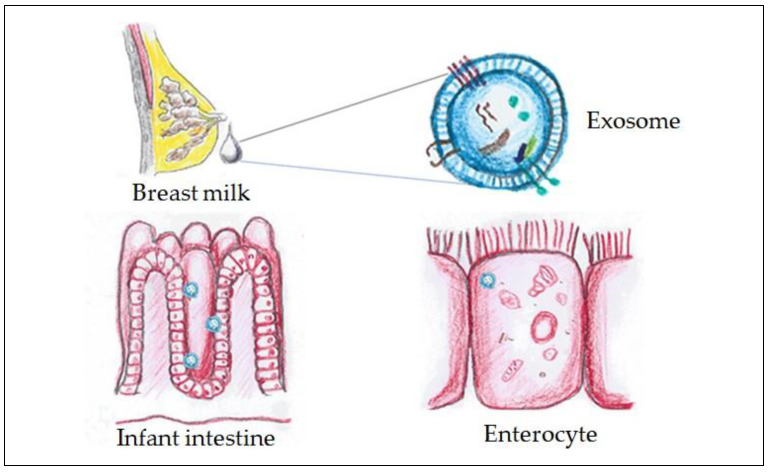
Exosomes present in breast milk contribute to the integrity of the intestinal barrier, improving epithelial cell cohesion and modulating the inflammatory response, which is crucial for the prevention of NEC (image created by the authors).

The development of a healthy gut microbiota is essential for the development of newborns, as it influences basic physiological functions such as nutrient digestibility, metabolism and immunity. During the first months of life, breast milk is the main source of microorganisms and bioactive factors that contribute to the development of the gut microbiota. In this context, breast milk exosomes play a key role in modulating the intestinal environment of the newborn [21] (Figure 1). Exosomes exert their effects by promoting the colonisation of beneficial bacteria, such as *Bifidobacterium* and *Lactobacillus*, which are essential for a balanced microbiome. These effects are mediated by the miRNAs present in exosomes, which regulate the expression of genes involved in intestinal cell proliferation and differentiation, promoting epithelial integrity and immunohomeostasis [22]. Exosomes also interact with human milk oligosaccharides (HMOs), which act as prebiotics to stimulate the growth of beneficial bacteria and create an environment conducive to microbial balance [23]. This is especially important in the context of early microbiota establishment, as an adequate gut colony has been shown to have long-term effects on infant health, including a reduced risk of inflammatory and infectious diseases [24].

Recent studies have shown that the presence of exosomes in breast milk is associated with a healthier microbial profile in infants [13]. In animal models, the administration of breast milk exosomes resulted in a significant increase in the diversity and abundance of beneficial gut bacteria, highlighting the critical role of these exosomes in modulating the gut microbiota [25]. Previous studies have shown that only breastfed term infants have specific health-promoting bacteria [26], and that breastfeeding has a more profound effect on the neonatal enterocyte microbiota genes that influence host defence and development compared to formula-fed term infants [26]. However, it is unknown how the composition of the gut microbiome of preterm infants is affected by the consumption of formula milk from mothers delivering preterm infants.

## 4. Exosomes and Protection Against Necrotising Enterocolitis (NEC)

Premature babies suffer from a number of complications due to the immaturity of their organs, which are poorly adapted to the extrauterine environment at birth. Preterm infants born before 32 weeks are particularly vulnerable. Preterm infants are at an increased risk of developing inflammatory bowel diseases, particularly necrotising enterocolitis. NEC affects approximately 10% of preterm infants weighing less than 1500 g and is one of the main factors of neonatal morbidity and mortality in the Spanish population [27]. Preterm infants are also often at risk of intestinal dysbiosis associated with caesarean section, maternal infection (e.g., chorioamnionitis), routine antibiotic treatment in the perinatal period and reduced exposure to breast milk. In conclusion, intestinal dysbiosis in preterm infants is problematic for both short- and long-term health and is considered an important risk factor for NEC [28].

Necrotising enterocolitis is a fulminant inflammatory disease that occurs chiefly in preterm infants and has a high mortality rate. The aetiology of NEC is associated with the interaction of multiple factors, which involve the immaturity of the gut barrier, dysbiosis and the hyperinflammatory response. Breast milk has been associated with a spectacular reduction in the incidence of NEC through the combined effects of immunological and prebiotic factors, such as exosomes [24].

Breast milk exosomes avert NEC through the promotion of intestinal barrier integrity. This is supported by upregulating the tight junction proteins ZO-1, claudin and occludin that promote epithelial cell cohesion and suppress the movement of pathogenic bacteria into the bloodstream [24,29]. In addition, miRNAs in exosomes can modulate the inflammatory response by inhibiting proinflammatory cytokine production, which is particularly useful for suppressing the excessive gut inflammation that characterises NEC [21].

Studies in animal models have shown that breast milk exosomes have a significant protective effect against NEC [30,31]. A recent study showed that mice that received breast milk exosomes prior to induction with LPS, a bacterial toxin that contributes to inflammation, developed less intestinal inflammation and had significantly lower mortality compared to those that did not receive exosomes [32].

**Figure 2:**
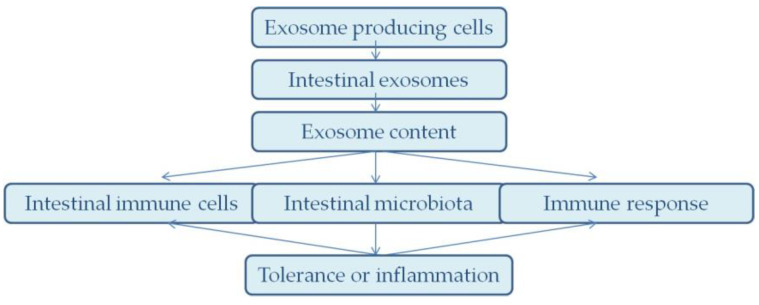
Diagram of the regulation of intestinal immunity by exosomes.

**Figure 3:**
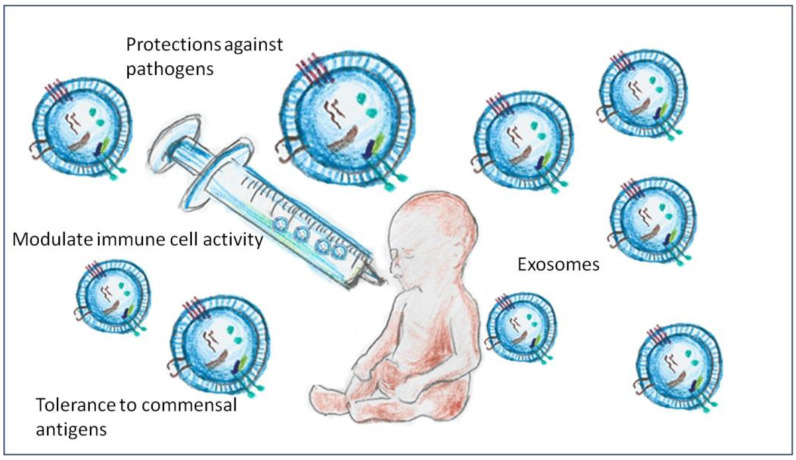
Exosomes modulate the activity of neonatal immune cells, promoting a balance between protection against pathogens and tolerance to commensal antigens, which is essential to avoid excessive inflammation (image created by the authors).

The immune system of the newborn is immature and in a rapid development process, which is significantly influenced by the components of breast milk (Figure 2). Exosomes play a key role in modulating the immune system, helping to balance the immune response between protection against pathogens and tolerance to non-pathogenic antigens (Figure 3). Exosomes contain miRNAs that regulate the expression of the key genes involved in immune cell activation and differentiation, allowing an appropriate response to infection while avoiding excessive inflammatory responses that can be harmful to the newborn [33].

One of the main mechanisms by which exosomes modulate the immune system is cytokine regulation. MiRNAs carried by exosomes, such as miR-148a and miR-30b, are involved in the inhibition of the production of proinflammatory cytokines, such as tumour necrosis factor alpha (*TNF-α*) and interleukin-6 (*IL-6*) [34]. This helps to reduce systemic and local inflammation in the gut, which favours an environment conducive to the infant’s immune development [13].

In addition, exosomes also promote the differentiation of T regulatory cells (Tregs), which play a crucial role in inducing immune tolerance and preventing autoimmune responses [35]. The presence of Tregs is essential to prevent the development of inflammatory diseases such as NEC and to promote a proper balance of the neonatal immune response [36].

Furthermore, several studies reveal that the proteomics of breast milk-derived exosomes promote immune and gastrointestinal system development and indirectly prevent necrotising enterocolitis by promoting the growth of beneficial bacteria such as *Bifidobacterium* and *Lactobacillus*, inhibiting intestinal pathogens and strengthening the epithelial barrier by increasing the expression of tight junction proteins. In addition, breast milk exosomes have been reported to modulate the immune response by reducing inflammation through the regulation of macrophages and dendritic cells and the production of anti-inflammatory cytokines (*IL-10*, *TGF-β*), and to protect intestinal epithelial cells (IEC) against H_2_ O_2_-induced cell toxicity [37]. This microbial regulation favours the production of protective metabolites such as butyrate, decreases bacterial translocation and reduces inflammasome activation and the proinflammatory response, creating a homeostatic intestinal environment that protects against NEC. These indicate that exosomes have therapeutic potential with respect to NEC.

Exosomes could indirectly prevent necrotising enterocolitis by modulating the intestinal microbiota, promoting the growth of beneficial bacteria and reducing opportunistic pathogens, although no studies have been conducted to prove this. However, at the experimental level, there is research on exosomes derived from mesenchymal stem cells that, when administered orally or intraperitoneally in animal models, modify intestinal dysbiosis, with observed increases in *Lactobacillus* and *Bifidobacterium* [37,38]

## 5. Techniques for the Isolation and Sequencing of Exosomes in Breast Milk

The isolation and sequencing of exosomes in milk represents a growing field of study due to interest in their role in intercellular communication, the transmission of biomolecules (such as proteins, lipids and RNA) and their potential use in biomarkers and treatments. The techniques used in exosome isolation must be highly efficient and capable of isolating exosomes from a variety of sample matrices. To test the quality of isolated exosomes, several techniques have been developed [39]: The ultracentrifugation process is considered the most frequent procedure and the ‘gold standard’ for exosome isolation [40]. It is based on the splitting of particles according to their size and density by centrifugal forces. For sample conditioning, milk is centrifuged at low speed (300–500 g) to remove cells and debris. Subsequently, it is centrifuged at 10,000–20,000× *g* in order to remove larger vesicles and undesirable particles, and ultrasonic centrifugation is carried out at a high speed (100,000–120,000× *g*) for exosome pelletisation (pellet by centrifugation). Finally, the pellet is resuspended, and ultracentrifugation is performed again to purify the exosomes. Although this technique provides good performance and scalability, it has some disadvantages, such as a lower purity compared to other methods as it may co-pelletise proteins, lipids and other non-exosomal components. It can also be detrimental to exosomes, which could compromise their integrity and bioactivity [39]. Another technique is polymer precipitation, which uses polymers such as polyethylene glycol (PEG) to form exosomes in solution. The first step is the polymer combination: PEG is added to the milk sample and left to incubate at 4 °C for several hours or overnight. Subsequently, evacuation is performed, where the exosomes are pelletised by centrifugation at a low speed (1500–10,000× *g*) [41]. This technique is simple, agile and does not require specialised machinery, although it has a reduced level of purification, since other proteins and particles are also precipitated. However, exclusion size chromatography (SEC) involves separating exosomes according to their size by using columns with particular-sized pores. The milk is processed in order to remove cells and debris, then the sample is passed through a SEC column and the exosomes are grouped into specific segments. The advantage here is the preservation and high purity of the exosomes for use in functional studies, therapeutic development or biomarker discovery [42]. SEC is a gentle method, ensuring that exosomes remain intact and functional, and is highly reproducible. This technique can be combined with other techniques and is particularly useful when a high purity is required for functional assays, therapeutic use and RNA/DNA analysis [43].

The most commonly used method to isolate exosomes according to their size is ultrafiltration. The milk sample passes through membranes with decreasing pores (such as 0.8 μm, 0.45 μm and 0.22 μm, respectively) and exosome concentrations are produced through ultrafiltration [44]. It is a simple and quick technique to perform. Immunoaffinity using antibodies specific for exosome surface proteins (such as CD9, CD63 or CD81) is also used to capture exosomes. First, antibody immobilisation is performed, where the antibodies are attached to a solid surface [43] (e.g., magnetic beads).

After exosome isolation, exosome capture is performed by incubating the milk sample with the antibody-coated beads. This is followed by elution, in which the exosomes are released from the beads.

Once isolated, the exosomes can be analysed for RNA, DNA or protein content. Techniques for RNA sequencing in exosomes include next-generation RNA sequencing (RNA-seq), which allows for the massively parallel sequencing of RNA contained in exosomes. First, total RNA is extracted from exosomes, then libraries are constructed where RNA is converted into complementary DNA (cDNA) and adapters are added for sequencing. In the latter, an NGS (next generation sequencing) platform is used to sequence the libraries. The sequencing data are aligned with reference genomes to identify and quantify RNAs. This technique allows for the identification of miRNAs, mRNAs and other non-coding RNAs in exosomes [45]. Another technique would be qPCR (Quantitative Polymerase Chain Reaction). This is a highly sensitive and specific technique to quantify specific RNAs in exosomes. RNA is extracted from the exosomes. RNA is converted to cDNA using reverse transcriptase and qPCR is performed using primers specific to the RNA of interest. It is used for the quantification of miRNAs or specific mRNAs in exosomes [46]. RNA microarrays, on the other hand, allow for the quantification of specific RNAs in exosomes by hybridisation with predefined probes. For RNA extraction, RNA is extracted from exosomes, then the RNA is labelled with fluorophores, where the labelled RNA is hybridised to a microarray containing specific probes [47].

The isolation of exosomes from milk is mainly performed by ultracentrifugation, polymeric precipitation or exclusion chromatography. Next-generation RNA sequencing (RNA-seq) is most commonly used to sequence their content, although qPCR and microarrays are also used for more detailed studies.

From the different techniques applied for the isolation and analysis of milk-derived exosomes, it is possible to obtain various parameters to characterise both their quality and biological composition. These parameters include the average size of the vesicles, their concentration (expressed as number of particles per millilitre of milk), their morphology and structural integrity, as well as the purity of the isolate in relation to other proteins present in milk. In addition, using molecular techniques, it is possible to identify specific exosomal markers (such as CD9, CD63 or CD81) and to analyse the internal content of these exosomes, including RNA profiles (such as microRNA and mRNA), bioactive proteins and, in some cases, lipids. These parameters, which are obtained directly from the exosomes present in milk, allow not only for their proper characterisation, but also the evaluation of their biological and functional potential in physiological, immunological or therapeutic processes [41,42,43,44,45,46,47].

## 6. Future Therapeutic Applications of Breast Milk Exosomes

Necrotising enterocolitis is one of the most devastating diseases of prematurity, with high morbidity and mortality, and there is an urgent need to develop effective treatments for this debilitating condition. Breast milk, known for its health benefits as it contains large amounts of exosomes, has been studied for decades and has the potential to treat NEC. Several studies have shown that breast milk reduces the incidence of NEC, although the condition of NEC is rare in infants whose diets contain breast milk. Breast milk feeding reduces the risk of NEC compared to formula milk, especially in preterm infants [48]. The therapeutic potential of breast milk-derived exosomes is emerging as a promising field of research in neonatology. Exosomes can be used to develop new intervention strategies for high-risk neonates, such as premature infants and those without access to fresh breast milk. Exosome replacement could improve gut health, modulate immune responses and reduce the risk of serious complications such as NEC [49,50].

The therapeutic development of bone marrow-derived extracellular vesicles (BM-EVs) for the treatment of necrotising enterocolitis has attracted considerable interest. Several animal models, such as rat, mouse and pig models, are widely used in laboratories to study this condition [51,52,53]. Using a rat model of NEC, human stem cell-derived extracellular vesicles have been shown to reduce the incidence of NEC in rat pups when administered orally in a dietary formula. The results suggest that oral administration of BM-EVs has a higher efficacy compared to the intraperitoneal route, which may be related to their stability and passage through the digestive tract [32,49].

One of the most prominent applications would be the incorporation of exosomes into infant formula or milk donated by milk banks [54]. Since pasteurisation procedures can affect the viability and functionality of exosomes, the enrichment of donated milk with isolated exosomes could ensure that infants receive the full immunological and protective effects of breast milk [24]. This would be especially useful in neonatal intensive care units (NICU), where premature infants are at increased risk of complications due to their immunological and digestive immaturity.

Another possible avenue for therapeutic intervention would be the direct administration of exosomes as therapy in neonates at high risk of developing NEC or other inflammatory diseases. Preclinical studies have suggested that exosomes may help to restore intestinal integrity and reduce inflammation, which may be essential for improving clinical outcomes in these patients [55].

In addition, research on the modulation of exosome production by dietary and maternal lifestyle interventions may open up new avenues to enhance the benefits of breast milk. Factors such as diet, health status and exposure to stressors have been shown to influence the composition of breast milk exosomes [56]. The optimisation of these factors may lead to a natural enrichment of exosomes and thus a greater transfer of benefits to the infant [57].

These previous findings suggest that breast milk exosomes may exert protective effects against necrotising enterocolitis and recent research has begun to explore whether exosomes present in the milk of other mammals could exert similar effects on intestinal epithelial cells. It has been reported that exosomes from rat milk markedly enhance the viability of intestinal epithelial cells, promote their proliferation and stimulate intestinal stem cell activity, which is reflected in the increased expression of the *Lgr5* gene [31]. In addition, other studies have analysed the possible benefits of exosomes in milk from other mammals, finding that porcine milk exosomes significantly promote the expression of *CDX2*, *IGF-1R* and *PCNA*, while inhibiting the expression of the *p53* gene, which is critical for intestinal proliferation [58]. In the case of bovine milk exosomes, they have been shown to increase goblet cell expression and mucin production and to decrease myeloperoxidase levels in experimental models of necrotising enterocolitis (NEC) [30]. These exosomes have also demonstrated protective effects against oxidative stress in *IEC-6* cells [30].

## 7. Challenges and Ethical Considerations

Despite the great therapeutic potential of breast milk exosomes, significant challenges remain before these interventions can be routinely introduced into clinical practice. One of the main challenges is the large-scale production of exosomes with homogeneous characteristics and properties. The extraction and isolation of exosomes from breast milk requires complex techniques, and the standardisation of these procedures is essential to ensure the efficacy and safety of exosome-based treatments [46,54].

It is also important to consider the ethical implications of using breast milk exosomes. Obtaining large quantities of breast milk for exosome production may affect the amount of milk available for breast milk banking, which may impact infants who rely on this source of nutrition. It is therefore essential to balance the research and development of new therapies with the need to ensure adequate access to breast milk for all infants.

## 8. Conclusions and Future Perspectives

Breast milk exosomes represent a new field of research in neonatology and infant health. They play an important role in modulating the immune system and in the development of a healthy gut microbiome in the newborn. Due to their ability to modulate cell differentiation, immune signalling and gut integrity, they have great potential for therapeutic interventions to improve the health of newborns, especially those at high risk.

The future of exosomes in neonatal medicine, especially for high-risk newborns such as preterm infants, is promising. Exosome-based therapies have the potential to transform the field of neonatal medicine by introducing new disease prevention mechanisms, such as mechanisms against NEC, and by improving long-term health outcomes for the most at-risk neonates. Future research should include human clinical trials to test the efficacy of exosome replacement and determine optimal doses to maximise benefits.

## Data Availability

Not applicable.
